# Anomalous Hall Conductivity and Nernst Effect of the Ideal Weyl Semimetallic Ferromagnet EuCd_2_As_2_

**DOI:** 10.1002/advs.202207121

**Published:** 2023-02-24

**Authors:** Subhajit Roychowdhury, Mengyu Yao, Kartik Samanta, Seokjin Bae, Dong Chen, Sailong Ju, Arjun Raghavan, Nitesh Kumar, Procopios Constantinou, Satya N. Guin, Nicholas Clark Plumb, Marisa Romanelli, Horst Borrmann, Maia G. Vergniory, Vladimir N. Strocov, Vidya Madhavan, Chandra Shekhar, Claudia Felser

**Affiliations:** ^1^ Max Planck Institute for Chemical Physics of Solids 01187 Dresden Germany; ^2^ Department of Physics and Materials Research Laboratory University of Illinois Urbana, Champaign Urbana IL 61801 USA; ^3^ Swiss Light Source Paul Scherrer Institute Villigen‐PSI CH‐5232 Switzerland; ^4^ S. N. Bose National Centre for Basic Sciences Salt Lake City Kolkata 700 106 India; ^5^ Department of Chemistry Birla Institute of Technology and Science Pilani ‐ Hyderabad Campus Hyderabad 500078 India; ^6^ Donostia International Physics Center Donostia‐San Sebastian 20018 Spain

**Keywords:** anomalous hall, ferromagnet, magnetoresistance, Nernst effect, Weyl semimetal

## Abstract

Weyl semimetal is a unique topological phase with topologically protected band crossings in the bulk and robust surface states called Fermi arcs. Weyl nodes always appear in pairs with opposite chiralities, and they need to have either time‐reversal or inversion symmetry broken. When the time‐reversal symmetry is broken the minimum number of Weyl points (WPs) is two. If these WPs are located at the Fermi level, they form an ideal Weyl semimetal (WSM). In this study, intrinsic ferromagnetic (FM) EuCd_2_As_2_ are grown, predicted to be an ideal WSM and studied its electronic structure by angle‐resolved photoemission spectroscopy, and scanning tunneling microscopy which agrees closely with the first principles calculations. Moreover, anomalous Hall conductivity and Nernst effect are observed, resulting from the non‐zero Berry curvature, and the topological Hall effect arising from changes in the band structure caused by spin canting produced by magnetic fields. These findings can help realize several exotic quantum phenomena in inorganic topological materials that are otherwise difficult to assess because of the presence of multiple pairs of Weyl nodes.

## Graphical abstract

Ferromagnetic EuCd_2_As_2_ exhibits anomalous Hall conductivity and Nernst effect, resulting from the non‐zero Berry curvature close to the Fermi level. Scanning tunneling microscopy and angle‐resolved photoemission spectroscopy are used to investigate its electronic structure which is in agreement with first principles calculations.

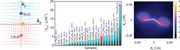

## 1 Introduction

Weyl semimetals (WSMs) have gained widespread interest because of their fundamental and technological importance in applications ranging from quantum computing to valleytronics.^[^ ^1^, ^2^ ^]^ In WSMs, quasiparticles at the topologically secured band crossings, that is, at nodal points, are governed by the Weyl Hamiltonian having linear dispersion;^[^ ^2^ ^]^ however, according to the Nielsen–Ninomiya “no‐go” theorem, Weyl nodes always appear in pairs with opposite chiralities.^[^ ^3^ ^]^ The surface states of WSMs are known as Fermi arcs, which connect the surface projection of Weyl points (WPs) with opposing chirality, but their number and position in momentum space are material specific.^[^ ^4^ ^]^ Although WSMs have much potential practical applications, finding unambiguous signatures of the Weyl physics and utilizing those for applications is challenging. Two main issues are: i) the existence of other non‐topological bands near the Fermi level;^[^ ^1^, ^5^ ^]^ and ii) multiple pairs of symmetrically unrelated Weyl points, which can impede the signatures of preferred Weyl physics.^[^ ^1^, ^2^ ^]^

**Figure 1:**
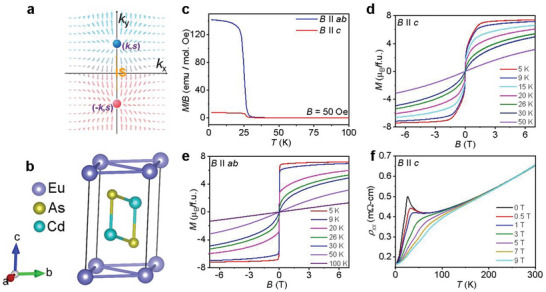
Crystal structure, resistivity, and magnetic measurements. a) Schematic of ideal WSMs with only two WPs in a magnetic system. Red and blue atoms represent WP 1 and WP 2, respectively; IS: inversion symmetry. b) Crystal structure of EuCd_2_As_2_; violet, yellow and blue atoms represent Eu, Cd and As, respectively. c) Temperature‐dependent field‐cooled magnetic susceptibility (*χ*) at *B* = 50 Oe for *B* || *ab* and *B* || *c*. Isothermal magnetization for d) *B* || *c* and e) *B* || *ab* at various temperatures. f) Variation in resistivity *ρ*_xx_, with temperature at various magnetic fields.

The WSM phase can be realized in crystals that break either time‐reversal symmetry (TRS) or inversion symmetry (IS), or both, in inorganic solids.^[^ ^1^, ^6^, ^7^, ^8^, ^9^, ^10^, ^11^ ^]^ In the case of broken IS, the minimum possible number of Weyl points is four; the popular Weyl semimetal TaAs contains a total of 12 pairs.^[^ ^9^, ^10^ ^]^ In stark contrast, if the time‐reversal symmetry is broken, it is possible to realize a minimum of two WPs; such a material can be designated an ideal WSM (**Figure** **1**a).^[^ ^1^ ^]^ Furthermore, broken‐TRS‐governed WSMs provide a platform for the interaction of magnetism and topological ordering, resulting in rich and unusual quantum phenomena such as quantum anomalous Hall (QAH) effects and axion insulators.^[^ ^1^, ^12^ ^]^ Thus, in these magnetic WSMs (MWSMs), in realizing Weyl physics such as chiral anomaly, chiral Majorana modes are more favored than WSMs with more WPs in which helicity is not well‐defined because of the broken Lorentz invariance.^[^ ^13^ ^]^

Recently, an angle‐resolved photoemission spectroscopy (ARPES) study revealed the characteristic features of Weyl phase, such as surface Fermi arcs and linear bulk band dispersions across the WPs, and confirmed the presence of TRS‐broken WSM phase in ferromagnetic (FM) Co_2_MnGa, Co_3_Sn_2_S_2_ and the pyrite CoS_2_^[^ ^11^, ^14^, ^15^ ^]^ Furthermore, Liu et al. observed large anomalous Hall conductivity in Co_3_Sn_2_S_2_ due to an enhanced Berry curvature contribution and its interaction with inherent magnetism, making MWSMs more attractive for potential applications.^[^ ^16^ ^]^ Bernevig and colleagues predicted theoretically a new series of Co‐based magnetic Heusler alloys that can behave as a Weyl system with several nodes having broken TRS.^[^ ^17^ ^]^ However, it is possible to realize two Weyl nodes near the Fermi level in this family with proper alloying only. Although Wang et al. recently realized an ideal WSM band experimentally in ultracold atoms by tailoring the 3D spin‐orbit coupling, realizing this observation requires special expertise.^[^ ^18^ ^]^ An intense search is underway for ideal magnetic Weyl materials to observe and control Weyl physics directly. However, the realization of an ideal WSM is still in its infancy. Therefore, researchers will continue to search for the same effect in solid‐state materials with easy synthesis techniques and air‐stable materials to tailor their properties.

Recently, EuCd_2_As_2_ (ECA), with a layered structure in which triangular Eu layers separate the Cd_2_As_2_ layers (space group of *P‐3m1*, Figure 1b), was proposed as an ideal MWSM, that is, featuring only two Weyl points.^[^ ^19^, ^20^ ^]^ ECA exhibits two different types of A‐type antiferromagnetic (AFM) ordering states below ≈9 K, that is, the FM spin of the triangular Eu layers might be along either the in‐plane or out‐of‐plane direction.^[^ ^21^, ^22^, ^23^ ^]^ Previous theoretical calculations predicted that ECA hosts the Dirac semimetal state with a single Dirac node near the Fermi level (*E*_F_) in the out‐of‐plane configuration and can transform to an MWSM with only two WPs, provided that *C*_3_ rotation symmetry is preserved in the spin configuration.^[^ ^21^, ^24^ ^]^ However, this threefold symmetry is no longer preserved in the in‐plane configuration, resulting in a gap opening of ≈1 meV, which turns into an exotic axion insulating state that is similar to MnBi_2_Te_4_^[^ ^21^, ^25^ ^]^ Boothroyd and co‐workers showed the evidence of a single WP pair only when a magnetic field larger than 1.6 T was applied along the *c*‐axis.^[^ ^19^ ^]^ Furthermore, ARPES measurements on the paramagnetic phase of EuCd_2_As_2_ (above the Neel temperature) established the existence of Weyl fermions due to the broken time‐reversal symmetry by FM‐like spin fluctuations.^[^ ^26^ ^]^

All of the abovementioned findings indicate that stabilizing the FM ground state in ECA is critical. Canfield and co‐workers were able to stabilize the FM state with a *T*_C_ of ≈26 K by tailoring the level of band filling in the system during synthesis, which has a strong impact on the magnetic ordering in the system.^[^ ^27^ ^]^ However, more recently Sanjeewa et al., also stabilized the FM phase with a similar reaction condition in Eu_1−x_Ba*_x_* Cd_2_As_2_.^[^ ^28^ ^]^ They corroborated the canting of out‐of‐plane Eu moments for the observation of FM spin configurations rather than the Eu vacancies.^[^ ^28^ ^]^ However, as of yet there is no experimental evidence for the canting of spin in the FM configuration. Previous studies provide limited and insufficient information on the magnetic, electrical and thermal transport behavior of FM‐EuCd_2_As_2_, which needs to be explored further in detail. This material presents an excellent opportunity for research because of its combination of magnetism and topology, as well as the indicator of Weyl fermions. The electronic structure obtained from the theory has shown similarity to previous reports; however, a deeper understanding of the Weyl physics in the FM phase is still missing.

In this study, we synthesized the FM phase of ECA using the salt flux method (as reported previously by Canfield and co‐workers^[^ ^27^ ^]^) and attempted to resolve previously unsolved puzzles associated with this spin configuration. We combined electrical, magnetic and thermal measurements with the ARPES and scanning tunneling microscopy (STM) followed by density functional theory (DFT) calculations to characterize its exotic transport properties.

## 2 Results and Discussion

To understand the stabilization of FM phase of EuCd_2_As_2_ in the present work by salt flux method (as reported previously in Ref. [27]), we have also prepared the AFM phase of EuCd_2_As_2_ by using Sn flux (as reported earlier in Ref. [21]). We have performed detailed structural analysis on both single crystals (AFM and FM). We have tabulated the parameters determined from Rietveld refinements of room temperature X‐ray diffraction data taken on single crystals of AFM and FM EuCd_2_As_2_ in Table S1, Supporting Information. From the refinements analysis, it is fair to say that our synthesized FM sample contains ≈1% Eu vacancy in the lattice whereas we have not observed any Eu deficiency for the AFM phase which is similar to the previous report by Canfield and co‐workers.^[^ ^27^ ^]^

Field‐cooled (FC) magnetic susceptibility measurements were performed on the *B* || *ab* (*χ_ab_*) and *B* || *c* (*χ_c_*) planes of ECA, with an applied field of 50 Oe. ECA exhibited a paramagnetic to ferromagnetic transition at *T* ≈ 26 K, similar to the results of prior works (Figure 1c).^[^ ^27^, ^28^ ^]^ However, the magnetic transition temperature is only ≈9 K for AFM‐EuCd_2_As_2_ crystal.^[^ ^21^ ^]^ Interestingly, the magnitude of *χ_ab_* is almost 13 times higher than that of *χ_c_* at 50 Oe, confirming the presence of substantial magnetic anisotropy in EuCd_2_As_2_ crystal. Figures 1d and 1e represent the magnetization isotherm data for the field parallel to both the *c* and *ab* planes, respectively, at temperatures ranging from 5 to 100 K. At an applied magnetic field of ≈0.2 T along the *ab* plane, magnetization saturated quickly at a value of 7.2 µ_B_ per formula unit at 5 K (Figure 1e). However, a large magnetic field of ≈1.4 T is required to saturate the magnetization when the field is applied along the *c* axis (Figure 1d). Thus, it is reasonable to conclude that the *ab* plane is the easy magnetic plane, and the *c* axis is the hard axis for magnetization in EuCd_2_As_2_. Spins of Eu^2+^ are responsible for the magnetism in this system. Temperature‐dependent resistivity data (*ρ*_xx_) exhibit a peak near 26 K where magnetic field *B* is applied parallel to the *c*‐axis, which is consistent with our magnetic susceptibility data (Figure 1c–f). We also observed a metamagnetic transition around 0.1 T when the applied field was parallel to the *c* axis. This result can be attributed to the increasing canting of spins towards the *c‐*axis with the applied field. We discuss later the effect of this spin canting in the electrical transport and further supported by DFT calculations.

**Figure 2:**
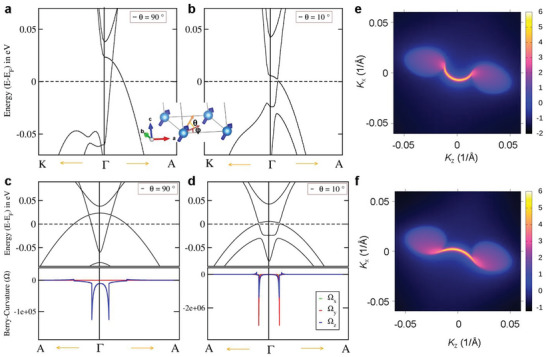
Theoretical calculation of noncollinear electronic structure and WPs. Band structure of ferromagnetic (FM) EuCd_2_As_2_ in GGA+*U*+SOC, zoomed along the (*K*–*Γ*–*A*) high symmetry line of the hexagonal Brillouin zone for magnetic moment along a) the *c*‐axis, that is, *θ* = 90^0^, b) *θ* = 10° canting. The canting angle “*θ*” is defined with respect to the *ab*‐plane, shown in inset and in the *ab*‐plane we considered the spin pointing in the diagonal direction, that is, *φ* = 45° in our calculations. c,d) Ferromagnetic band structure of EuCd_2_As_2_ zoomed into the locations of the Weyl point in comparison with the computed Berry curvature along the high symmetry line of *A*–*Γ*–*A* for the magnetic moment along c) the *c*‐axis, that is, *θ* = 90°, d) *θ* = 10° canting. (e) and (f) Fermi arcs connecting the projected WPs on the top and bottom (101) surfaces, respectively, of EuCd_2_As_2_ in the FM state with magnetic moment along the *c*‐axis.

To gain a deeper understanding of the electronic structure of the FM‐EuCd_2_As_2_ system, we carried out detailed first‐principles DFT calculations, which are helpful to understanding the electrical transport of the system (**Figure** **2**, and Figures S8 and S9, Supporting Information). A localized magnetic moment at the Eu 4*f* site was found to be 6.9 µ_B_, consistent with the half‐filled 4*f* (S = 7/2) states. Orbital magnetic moment is completely quenched and essentially results in zero orbital magnetic moment because of the half‐filled *f*‐orbitals. The electronic band structure was calculated with the generalized gradient approximation (GGA)+Coulomb interaction strength (Hubbard *U*)+spin‐orbit coupling (*SOC*) considering the Eu 4*f* spins fully spin‐polarized along the *c*‐axis. We found a semi‐metallic ground state with Cd‐5*s* and As‐4*p* states dominating around the Fermi energy. Figure S8a, Supporting Information, shows the GGA+*U* (5 eV)+*SOC* band structure (green colors) in comparison with Wannier bands (red colors) close to the *Γ*–*A* direction of the hexagonal Brillouin zone. Around the *Γ* point, we observed a band crossing and a large contribution of the Berry curvature (Figure S8b, Supporting Information). The FM configuration, with Eu 4*f* spin aligned along the *c* axis, lifts the degeneracy of the bands, and we observed a single pair of Weyl nodes along the *Γ*–*A* high symmetry line at the wave vectors **k** = (0.000, 0.00, ± 0.027 1/Å). The corresponding single pair of Weyl points connecting these two wave vectors is shown in Figure S8c, Supporting Information. More importantly, we obtained a single pair of Weyl points without the presence of any additional trivial or nontrivial bands near the Fermi level, satisfying the condition of an ideal Weyl semimetal (Figure 1a). When we projected the single pair of Weyl points onto the crystalline (101) surface of EuCd_2_As_2_, open Fermi arcs connecting the projected Weyl points were observed. Figures 2e and 2f show the open Fermi arcs on both the top (Figure 2e) and bottom (Figure 2f) (101) surfaces of EuCd_2_As_2_.

Spin orientation contributes to the evolution of band structure, which causes band inversions and gap opening at certain canting angles, resulting significant augmentation of Berry curvature in the system. Recently, Parker and co‐workers have shown the influence of spin canting on the electronic structure and Weyl physics via Neutron scattering and DFT calculations.^[^ ^29^ ^]^ Now, we have also calculated the electronic structure of FM‐EuCd_2_As_2_ by considering several canting angles (Figure 2a–d, and Figure S9, Supporting Information). From DFT calculations, we observed that band structure is extremely sensitive to canting angle, influencing the Weyl physics even at very small canting angles. From our initial calculations we observed that a single pairs of Weyl point lies ≈20 meV above the Fermi level. However, the position of WPs moves closer to the Fermi level along *Γ*–*A* direction of Brillouin zone (BZ), Table S2, Supporting Information. With a small canting angle of 10^0^, we realize WP resides closely to the Fermi level (above ≈2 meV), resembles to the ideal Weyl physics.

**Figure 3:**
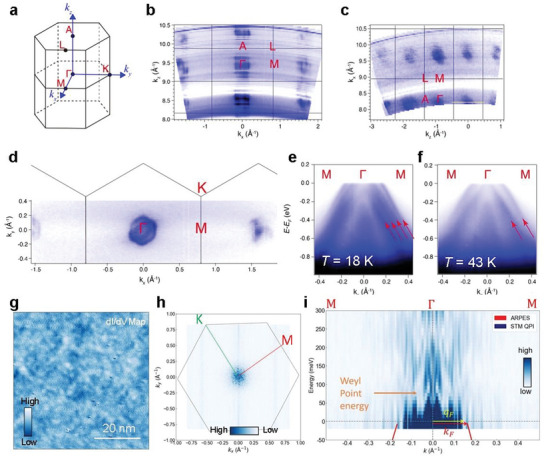
Spectroscopic data of EuCd_2_As_2_. a) Bulk BZ of EuCd_2_As_2_ with high‐symmetry points labeled. b) Out‐of‐plane Fermi surface mapping within the *k_x_*–*k_z_* plane, acquired with photon energies from 240 to 420 eV. c) Out‐of‐plane constant energy mapping at *E*_B_ = 0.1 eV within the *k_z_*–*k_x_* plane, acquired with photon energies ranging from 250 to 420 eV. d) In‐plane Fermi surface mapping acquired with *hν* = 270 eV. (e) and (f) ARPES intensity plots along the *Γ*–*M* direction measured at 18 and 43 K, respectively. g) Spatially resolved d*I*/d*V* conductance map. h) Fourier transform of d*I*/d*V* conductance map for wide range of *k*‐space (−1.0 to 1.0 Å^−1^) at 0 mV. Brillouin zone is marked by a gray hexagon. i) Energy‐momentum linecut of the quasiparticle interference (QPI) along the *Γ*–*M*. The filled region of the QPI signal originates from the scattering from the bulk bands, showing their dispersion. The orange arrow displays the expected Weyl point energy (based on extrapolation of the dispersion from Figures S10a and S11b, Supporting Information). The red solid curve is the dispersion from the ARPES along the *Γ*–*M* direction (Figure 3e). The dark blue intensity plot represents QPI signal. The yellow arrow represents dominant the scattering wavevector at Fermi energy (*q*_F_) which is similar in the length to the Fermi wavevector (*k*_F_) displayed in the red arrow.

To investigate the topological features, we performed systematic experimental electronic structure studies on both the *Γ*–*M*–*K* plane and the *Γ*–*K*–*A* plane using synchrotron‐based ARPES with soft X‐ray photons (**Figure** **3**a–f, and Figures S10–S12, Supporting Information). The increase in the photoelectron mean free path with photon energy reduces the intrinsic uncertainty of the out‐of‐plane wave vector *k*_z_, allowing accurate determination of the 3D band structure.^[^ ^30^ ^]^ The high‐symmetry points and coordinates are shown in Figure 3a. The EuCd_2_As_2_ material has a natural cleaving direction along the *c*‐axis. Fermi surface mappings within the *k*_x_–*k*_z_ and *k*_x_–*k*_y_ planes were carried out, as shown in Figure 3b–d. A series of Fermi pockets assembled by hole bands are located at the center of the Brillouin zone (BZ). In order to analyze the effect of magnetic order on the band structure, we obtained the ARPES spectrum along the *M*–*Γ*–*M* direction at two different temperatures. By comparing the ARPES spectra taken at 18 K (Figure 3e) and 43 K (Figure 3f) with *hν* = 270 eV, which are in the FM and paramagnetic (PM) states, respectively, we observed significant differences in the band structure. Therefore, we were able to detect the FM phase transition and band structure evolution with temperature in EuCd_2_As_2_ by ARPES. The degeneracy of the bands was lifted by the FM interaction, which caused a band splitting (Figure 3e).

Similar changes were also observed along the *Γ*–*A* direction. By cleaving the side surface of the sample, we were able to measure the band structure along the *Γ*–*A* direction in high resolution, which has not been studied earlier in FM‐EuCd_2_As_2_ samples. Because of the limited size of the sample piece, the spectra quality of the side surface was not as good as that of the top surface. However, by plotting the curvature images, band splitting was still observed in the FM phase, as indicated by the red arrows in Figure S10b, Supporting Information; the band splitting disappeared and degenerated into one in the PM phase (Figure S10d, Supporting Information). We have also performed ARPES experiment on another crystal of FM‐EuCd_2_As_2_ at 18 K with 126 eV and overlaid the ARPES intensity plot with the calculated band structure (Figure S11b, Supporting Information). EuCd_2_As_2_ band dispersion measured by ARPES shows reasonable agreement with DFT calculations. Moreover, these ARPES results reveal the evolution of the band structure affected by the magnetic structure and exhibit close agreement with our first‐principle calculations.

EuCd_2_As_2_ split into majority and minority spin bands as a result of FM transition.^[^ ^31^ ^]^ By comparing the ARPES spectra taken at 18 K (Figure 3e) and 43 K (Figure 3f), which are in the FM and paramagnetic (PM) states, respectively, we observed significant differences in the band structure. This is consistent with our DFT calculations and previously report by Jo et al.^[^ ^31^ ^]^ Earlier in PM‐EuCd_2_As_2_, the splitting of energy bands was observed due to FM fluctuations that were spatially correlated.^[^ ^26^ ^]^ According to our calculations, the distance between two Weyl point is about ≈0.05 Å, which is very hard to resolve in ARPES spectra. We observed cone like band dispersion below *E*_F_, in correspondence with our DFT calculations.

We shifted the chemical potential in the calculations downward by roughly few hundreds meV in FM states. These observations are compatible with the evidence of Eu vacancies from single crystal X‐ray refinement analysis (Table S1, Supporting Information). Therefore, doping level is important for the FM to expect the Weyl points. At our present carrier concentration (≈10^20^ cm^−3^), chemical potential is ≈100 meV far away from the Fermi level. To access the Fermi level and electron band, less carrier concentration is required. Our system is highly hole doped due to the intrinsic Eu vacancy. Therefore, further electron doping in the system will be helpful to reduce the carrier density.

Moreover, we have also plotted the momentum‐distribution curves of FM and PM‐EuCd_2_As_2_ along *M*‐*Γ*‐*M* direction to visualize the band splitting in the respective data and suppression of the scattering effect at the FM transition (Figure S12, Supporting Information). In response to decreasing temperatures, Zeeman splitting becomes stronger as the internal field increases (i.e., the FM order parameter).^[^ ^31^ ^]^ Therefore, splitting decreases with increasing temperature and disappears at FM transition temperatures.

To reveal the electronic structure above Fermi energy (*E*_F_), we conducted Fourier transform scanning tunneling spectroscopy (FT‐STS) and visualize quasiparticle interference. Spatially resolved dI/dV conductance maps at various energies were taken on the cleaved *ab*‐plane surface (Figure 3g). The spatial modulation in these maps is due to the interference between quasiparticle scattering. FT of the maps (FT‐STS) allows one to extract the scattering vectors at various energy, which in turn provide information on band dispersion.

Figure 3h shows a representative FT‐STS image at 0 meV. A linecut of the FT image along the *Γ*‐*M* is displayed in Figure 3i. The filled intensity (dark blue) is typically due to scattering within bulk bands and represents their dispersion. In this case, the length of the maximum scattering wavevector should be twice as large as that of the wavevector of the bulk band ($|\vec{q}\left|=2\right|\vec{k}|$), which corresponds to the scattering from one side to the other side of the bulk band ($\vec{k}$ to ‐$\vec{k}$). However, our observation in this material is that the maximum scattering vector at the Fermi energy is equal to the Fermi wavevector ($|{\vec{q}}_{F}\left|=\right|{\vec{k}}_{F}|$) as displayed in Figure 3i. Note that the $|{\vec{k}}_{F}|$ is obtained by the ARPES data (Figure 3e). This observation implies there exists dominant scattering between the bulk bands and states near the *Γ* point.

A possible origin of the observation could be the scattering between the bulk band along the *M* direction and Fermi arc states projected densely to the *Γ* point at the *k_x_*‐*k_y_* plane. As seen from Figure 2e,f, the DFT result shows that Weyl nodes and Fermi arc states exist within 0.02 Å^‐1^ from the *Γ* point, and the arc states have a finite (≤ 0.01Å^‐1^) in‐plane component. This is much smaller than in‐plane *k*_F_ ≈ 0.2Å^‐1^. Thus, the *k_x_*‐*k_y_* plane projection of the arc states is a line (continuum) of states highly condensed near the *Γ* point. As illustrated in Figure S14a, Supporting Information, one can imagine that the scattering from these densely located projected arc states at *Γ* point to a one bulk band state share nearly the same $\vec{q}$. The intensity in FT‐STS of these nearly degenerate scatterings could be much larger than the intensity of the scattering from one bulk band state to the other at opposite momentum which is unique. Note that this “half” scattering vector is repeatedly observed for another momentum direction (*K* point) and the other sample (sample 2 in Figure S13e, Supporting Information).

We conducted electrical and thermal transport measurements on the FM‐EuCd_2_As_2_ crystal in order to understand how its topological states affect its physical properties. As the temperature decreased, the *ρ*_xx_ decreased, demonstrating that the compound is metallic in nature (Figure 1f). Our studies focused on the Hall resistivity (*ρ*_yx_) and ordinary resistivity (longitudinal and transverse) (*ρ*_xx_) of EuCd_2_As_2_ at different temperatures as a function of field *B*.

**Figure 4:**
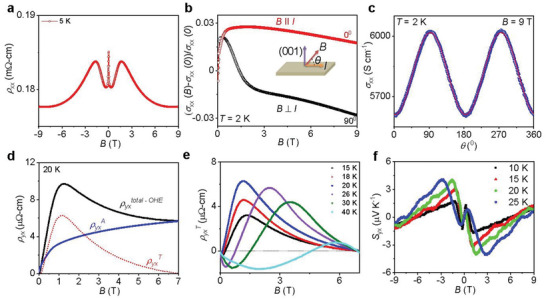
Electrical and thermal transport measurements a) Field‐dependent resistivity (*ρ*_xx_) of EuCd_2_As_2_ at 5 K. b) Field dependence of magnetoconductivity [*σ*_xx_(*B*) − *σ*_xx_(*0*))/*σ*_xx_(*0*)] at 2 K for *B* ⟂ *I* and *B* // *I* configuration. Inset shows the measurement set up. c) Angle, *θ* dependent electrical conductivity at 2 K and 9T. d) Field dependent decoupling of total Hall resistivity to anomalous Hall resistivity, $\left(\rho_{\mathrm{yx}}^{A}\right)$, and topological Hall resistivity, $\left(\rho_{\mathrm{yx}}^{T}\right)$ at 20 K. OHE represent ordinary hall effect. e) Field‐dependent $\rho_{\mathrm{yx}}^{T}$ at various temperatures. f) Field dependent Nernst thermopower (*S*_yx_) at various temperatures.

The resistivity peak enhances with temperature up to the magnetic transition temperature, *T*_C_ and is then decreases at higher temperatures, similar to previously reported for AFM samples (**Figure** **4**a and Figure S1, Supporting Information).^[^ ^19^ ^]^ At 5 K, initially a small drop in *ρ*_xx_ was observed in the low‐field regime (*B* ≤ 0.15 T), possibly owing to the decrease in spin scattering. Below *T*_C_, small drop in *ρ*_xx_ till exist which is in agreement with the FM structure of EuCd_2_As_2_. Secondly, it was observed that a peak in *ρ*_xx_ occurs in the middle regime (0.15 T ≤ *B* ≤ 1.60 T). This enhancement in resistivity can be attributed to the increased canting of spins towards the *c*‐axis with applied field. With increasing temperature, field strength corresponding to this peak decrease and finally vanishes above *T*c. Moreover, in the high‐field region (*B* > 1.6 T), spins were completely polarized.

Transverse magnetoresistance (TMR) measurements are shown in Figure S3a, Supporting Information, in which *B* || *c* and *I* || *ab* (i.e., *B* ⟂ *I*; MR = [(*ρ*(*B*) − *ρ*(*0*)]/*ρ*(*0*)). We observed a maximum negative magnetoresistance (nMR) of ≈54% at 26 K, which is close to the Curie temperature of the material. We also measured the longitudinal magnetoresistance (LMR) at different temperatures in which *B* || *I* (Figure S3b, Supporting Information). In this configuration as well, we observed a large nMR of ≈58%, slightly higher than that of the other configuration. Weyl materials are generally believed to have a nMR with this configuration (*B* || *I*) because of chiral anomalies. However, we observed negative MR for both the configuration for the temperature range of 5 K to 50 K which is commonly observed in a ferromagnetic system because of the field driven suppression of spin fluctuations (thermal). Upon warming, suppression of spin fluctuation becomes stronger with maximum around *T*_C_ (≈26 K). Above *T*_C_, it begins to become shallower and eventually turns into a regular parabolic shape about 50 K.

We used angle dependent magnetoresistance measurements to demonstrate the chiral anomaly, which adds to the evidence for the existence of Weyl fermions around the Fermi energy. When the electric current, *I* and magnetic field, *B* are parallel in Weyl metals, the numerical imbalance between the Weyl nodes with opposite chirality is expected to create a negative longitudinal magnetoresistance (LMR; *B* // *I*), while the transverse magnetoresistance (TMR; *B* ⟂ *I*) stays positive (Figure S2, Supporting Information).^[^ ^16^ ^]^ However, it is difficult to observe such behavior in a ferromagnetic system at weak *B* due to the presence of magnetic fluctuation which results positive magnetoconductance for any angle between *B* and *I*. Therefore, to reduce the effects of magnetic fluctuations, measurements must be carried out at sufficiently low temperatures and in the presence of sufficiently strong magnetic fields. Similar behavior was observed previously in case of another ferromagnetic MWSM Co_2_MnGa.^[^ ^32^ ^]^

We have shown the field dependent longitudinal magnetoconductivity (LMC) and transverse magnetoconductivity (TMC) at 2 K with different angle between *B* and *I* (Figure 4b and Figure S3c, Supporting Information). Importantly, we observe positive LMC (*B* // *I*) and negative TMC (*B* ⟂ *I*) and a cos^2^(*θ*) dependence where *θ* is the angle between *B* and *I* (Figure S4c, Supporting Information) at 9T similar to Co_2_MnGa.^[^ ^32^ ^]^ Thus, our transport data clearly identifies the chiral anomaly, indicating the presence of Weyl fermions in FM‐EuCd_2_As_2_.

Besides chiral anomaly, current jetting effect can also cause positive LMC. This current jetting effect has recently been proven to be the primary cause of the positive LMC reported in some Weyl semimetals.^[^ ^33^ ^]^ This effect can be seen in high mobility compensated Weyl semimetals like NbP, NbAs, TaAs, and TaP where the sample's extremely non‐uniform current distribution causes positive LMC.^[^ ^33^ ^]^ We can easily exclude this effect on our sample because the carrier mobility is quite small in FM‐EuCd_2_As_2_ compared to NbP, NbAs, TaAs and TaP. However, we have performed more experiments to exclude the current jetting effect. We observed similar resistivity for two different voltage contact on the sample at *B* // *I* configuration up to 9T (Figure S3d, Supporting Information) which confirms the negligible contributions of current jetting effect in FM‐EuCd_2_As_2_.

Then, we focused on the Hall resistivity (*ρ*_yx_) of single‐crystalline EuCd_2_As_2_. Figure 4d and Figure S4a, Supporting Information illustrate the Hall resistivity with an applied magnetic field at different temperatures ranging from 5 to 100 K. *B* || *c* and *I* || *ab* is the measurement configuration for *ρ*_yx_. Typically, a magnetic system describes *ρ*_yx_ as having three separate contributions, namely, *ρ*_yx_ = *R*_0_µ_0_ *H* + *R*_S_ *M* + *ρ*_yx_^T^.^[^ ^34^ ^]^ The first three terms denote ordinary, anomalous, and topological resistivities, respectively. *ρ*_yx_ exhibits anomalous behavior up to ≈30 K, as shown in Figure S4a, Supporting Information. We separated the anomalous and topological Hall contribution from the total Hall resistivity according to the above equation, as shown in Figure 4d,e and Figure S4b, Supporting Information. We have plotted anomalous Hall resistivity, *ρ*_yx_^A^ with variation of temperature in Figure S5a, Supporting Information. The anomalous Hall resistivity value is ≈2.7 µΩ‐cm at 2 K. *ρ*_yx_^A^ values increases with temperature. A maximum *ρ*_yx_^A^ of ≈4.28 µΩ‐cm was observed at 26 K which is close to the ferromagnetic transition temperature (*T*_C_). Interestingly, we observe finite *ρ*_yx_^A^ at *T* > *T*_C_ (Figure S5a, Supporting Information). The presence of finite anomalous Hall resistivity above *T*_C_ can only be attributed to Berry curvature anomalies in momentum‐space, such as Weyl points. In the paramagnetic phase, magnetic moments are distributed randomly and uniformly within each domain but are the same within each domain. As a result of the breaking of time‐reversal symmetry, Weyl points appear in each of the ferromagnetic domains. The polarity of each pair of Weyl points is compensated, however, on a macroscopic level, by a pair in another domain with an opposite magnetization. This results in the cancellation of both positive and negative contributions to the transverse transport. As a result, there is no spontaneous component to the anomalous Hall effect above *T*_C_. Therefore, when we applied an external magnetic field along the *c*‐axis, domains with moments along the *c*‐axis are promoted, resulting in a higher probability of Weyl points in the *Γ*–*A* line. Finite AHE results from all Weyl point pairs having nonzero net polarity.

We estimated the anomalous Hall conductivity $\sigma_{\mathrm{xy}}^{A}$ from the measured *ρ*_yx_ and *ρ*_xx_ values by using the equation $\sigma_{\mathrm{xy}}^{A}=\frac{\rho_{\mathrm{yx}}}{\rho_{\mathrm{yx}}^{2}+\rho_{\mathrm{xx}}^{2}}$.^[^ ^16^ ^]^ A maximum anomalous Hall conductivity of ≈100 Scm^−1^ and anomalous Hall angle ($\frac{\sigma_{\mathrm{xy}}^{A}}{\sigma_{\mathrm{xx}}}$) of ≈1.6% was observed at 2 K (Figure S6a, Supporting Information). Furthermore, we fitted the $\rho_{\mathrm{yx}}^{A}$ data to the *ρ*_xx_ value of EuCd_2_As_2_ in order to understand the origin of the anomalous Hall effect (intrinsic or extrinsic) in the present system (Figure S5b, Supporting Information). We observed a linear fitting in the $\rho_{\mathrm{yx}}^{A}$ versus $\rho_{\mathrm{xx}}^{2}$ plot in the low‐temperature regime, resulting in an intrinsic anomalous Hall conductivity of ≈42 Scm^−1^. Furthermore, with the constructed tight binding Hamiltonian considering the atomic orbital‐like maximally localized Wannier functions of Eu‐*s*, *d*, *f*, Cd‐*s*, *p* and As‐*p* states, we calculated the intrinsic contribution of the anomalous Hall effect (AHC); at *E*_F_, the value of AHC was found to be ≈36 Scm^−1^ (Figure S6b, Supporting Information). Thus, it is reasonable to conclude that the AHC in the FM‐EuCd_2_As_2_ has an intrinsic contribution resulting from the Berry curvature. Similar behaviors have also been observed previously in ferromagnetic Weyl semimetal Co_3_Sn_2_S_2_.^[^ ^16^ ^]^

At *T* > 50 K, *ρ*_yx_ becomes linear up to 9 T, like a normal conductor. Based on the Hall resistivity sign, holes in EuCd_2_As_2_ are the majority charge carriers. According to the single‐band model, the carrier concentration of the present sample is ≈1.04 × 10^20^ cm^−3^ at 100 K.

Apart from the anomalous Hall resistivity, another important feature observed in the present system is the presence of hump‐like behavior in the Hall resistivity data (Figure 4d). However, we did not observe such features in our magnetization data, which suggests the presence of an extra contribution to the total Hall resistivity, known as the topological Hall effect (THE).^[^ ^34^ ^]^ We observed a maximum topological Hall resistivity ($\rho_{\mathrm{yx}}^{T}$) of ≈6 µΩ‐cm at 20 K, which is higher than that of the well‐known noncollinear Mn_3_Sn, LaMn_2_Ge_2_, YMn_6_Sn_6_ and Gd_2_PdSi_3_ systems.^[^ ^35^, ^36^, ^37^, ^38^ ^]^ We have summarized the main observations for temperature variation for field dependent topological Hall resistivity (Figure 4e and Figure S7, Supporting Information) as follows: i) when the temperature of the FM and PM phases increases, the width of the positive peak widens unlike the AFM samples;^[^ ^39^ ^]^ ii) positive peaks increase in amplitude with temperature up to 20 K, then decrease, whereas the amplitude of the negative peak increases up to 40 K; iii) the negative peak is only observed in the PM phase like the AFM samples;^[^ ^39^ ^]^ and iv) the field value corresponding to the positive maxima enhances with increasing temperature in FM phase. Interestingly, in the case of AFM phase of EuCd_2_As_2_, amplitude, field value and width decrease with increasing temperature, whereas we found all three parameters increasing with temperature in the case of FM phase.

Recently, Sanjeewa et al. demonstrated that substitution of Ba in EuCd_2_As_2_ leads to canting of Eu spins.^[^ ^28^ ^]^ However, they did not observe any signature of the spin‐canting from the electrical transport data. They provided little information about their pristine sample and claimed that canting is responsible for the stabilization of EuCd_2_As_2_ in the FM phase instead of the AFM phase in this synthesis condition. Recently Taddei et al. have performed the detailed neutron diffraction study on FM‐EuCd_2_As_2_ (magnetic space group *C*2′/*m*′) and confirmed the Eu moments pointing along the [210] direction in plane and canted ≈30° out of plane.^[^ ^29^ ^]^ Interestingly, we have observed topological Hall signal in our resistivity measurement which was not observed in the previous study.^[^ ^28^ ^]^

The THE can have different types of origins: I) one possibility is that THE is due to the topology of the material's magnetization texture, which is related to its real space picture; and II) another possibility is the THE is caused by a reciprocal band structure involving Weyl nodes, known as momentum space picture.^[^ ^40^ ^]^

The spatial inversion in EuCd_2_As_2_ is a lattice symmetry with point group $\bar{3}$ *m*1. Moreover, any midpoint between neighboring Eu atoms will always be an inversion center, thus preventing the presence of the Dzyaloshinskii–Moriya interaction. We thus conclude that there is no real‐space Berry phase in this system and the observed THE does not correspond to a real‐space THE, similar to AFM‐EuCd_2_As_2_ sample.^[^ ^39^ ^]^

Basically, momentum space scenarios rely on the Weyl nodes, which act as both sources and sinks of Berry curvature.^[^ ^19^ ^]^ In this case, the THE is proportional to the Brillouin zone integration of the Berry curvature.^[^ ^40^ ^]^ We can therefore make the same observation since the Berry phase is related to the Berry curvature through closed‐path integration, and we can conclude that an electron's reaction to a pseudo‐field caused by a Weyl node cannot be differentiated from an electron's reaction to an external magnetic field. This explains the existence of the THE as the result of Weyl nodes.

Moreover, we observed a linear fitting in the $\rho_{yx}^{T}$ versus $\rho_{xx}^{2}$ plot in the low‐temperature regime (*T* < *T*_C_), providing evidence for a momentum‐space origin for the THE in FM‐EuCd_2_As_2_ (Figure S7b, Supporting Information). Similar results were also observed previously in AFM‐EuCd_2_As_2_.^[^ ^39^ ^]^

From our DFT calculations as discussed earlier, we observed that band structure is extremely sensitive to canting angle, influencing the Weyl physics even at very small canting angles. The position of WPs moves closer to the Fermi level along *Γ*–*A* direction of Brillouin zone (BZ) with a small canting angle of 10°. In addition, this provides another indication of the importance of the spin canting for the tuning of the band structure. Therefore, our results confirm that the THE‐like feature of EuCd_2_As_2_ is intrinsic and results from changes in the band structure caused by spin canting caused by magnetic fields.

Thermoelectrical transport is also a source of information since it is sensitive to changes in the Berry curvature at the Fermi level. We measured Nernst effect for the first time for FM‐EuCd_2_As_2_ as per our knowledge. It is well known that a ferromagnetic material generates an anomalous transverse voltage mutually perpendicular to both heat current and magnetization demonstrates the so‐called anomalous Nernst effect (ANE).^[^ ^32^ ^]^ ANE observed in several noncollinear antiferromagnets and nonmagnetic topological materials due to the presence of net Berry curvature near the Fermi level.^[^ ^41^ ^]^ In Weyl semimetals, Weyl points act as a source of Berry curvature, resulting large ANE. Previously, Xu et al. studied the Nernst effect of AFM‐EuCd_2_As_2_ where they observed ANE at both AFM and PM region due to the presence of Weyl point resulting from FM fluctuations in the system.^[^ ^39^ ^]^ Here, we measure the Nernst effect for the FM‐EuCd_2_As_2_ system which is predicted to be an ideal Weyl semimetal (Figure 4f). The Nernst thermopower also shows contributions from both ordinary and anomalous Nernst thermopower, similar to the Hall data. The measured Nernst thermopower, *S*_yx_ in magnetic system is usually defined as a combination of two terms:^[^ ^39^ ^]^ *S*_yx_ = *S* ^O^ + *S* ^A^ The anomalous contribution is separated from the total Nernst thermopower and is presented in Figure S16, Supporting Information.

**Figure 5:**
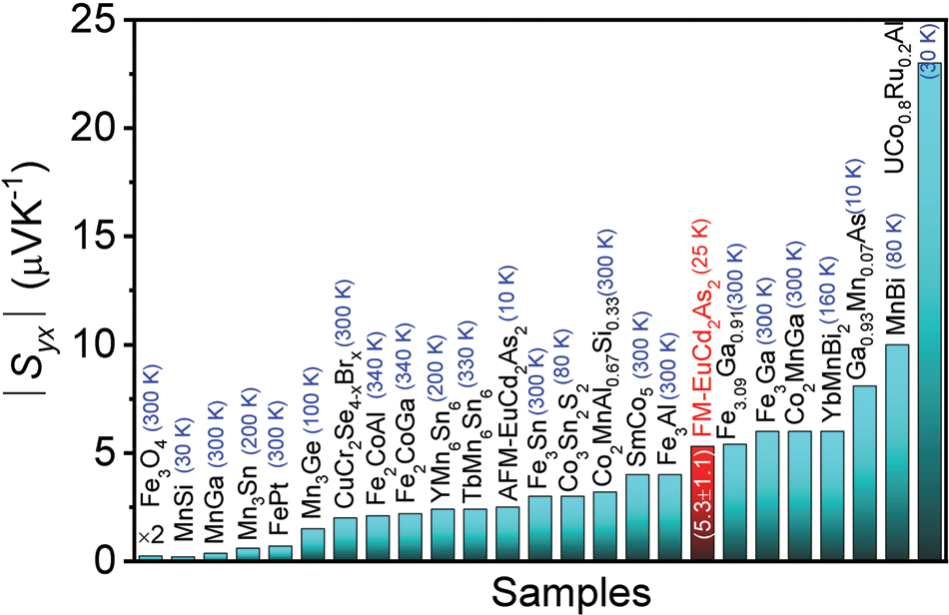
Comparison of thermal transport data. Comparison of anomalous Nernst thermopower between EuCd_2_As_2_ in the present study and previously reported ferromagnet, antiferromagnet materials.^[^ ^32^, ^39^, ^41^, ^42^, ^43^, ^44^, ^45^, ^46^, ^47^, ^48^, ^49^, ^50^, ^51^, ^52^, ^53^, ^54^, ^55^, ^56^, ^57^, ^58^, ^59^, ^60^ ^]^

It is expected to observe large ANE in the FM phase of EuCd_2_As_2_ compared to AFM phase due to the large contribution of Berry curvature near the Fermi level arising from two Weyl points. Importantly, we observe a large ANE of ≈5.3 ± 1.1 µV K^−1^ at 25 K, close to the FM transition temperature (*T*_C_ ≈ 26 K) (Figure S16, Supporting Information). We plotted and compared anomalous Nernst thermopower for FM‐EuCd_2_As_2_ with AFM‐EuCd_2_As_2_, other reported ferromagnets and non‐collinear antiferromagnets (**Figure** **5**).^[^ ^32^, ^39^, ^41^, ^42^, ^43^, ^44^, ^45^, ^46^, ^47^, ^48^, ^49^, ^50^, ^51^, ^52^, ^53^, ^54^, ^55^, ^56^, ^57^, ^58^, ^59^, ^60^ ^]^ FM‐EuCd_2_As_2_ exhibits large anomalous Nernst signal because the Weyl points lies close to Fermi level.

## 3 Conclusion

In summary, we investigated magnetic, electrical, and thermal transport of FM‐EuCd_2_As_2_. In addition, we studied its electronic structure by ARPES, and STM which agrees closely with DFT calculations. Anomalous Hall conductivity and Nernst effect in this material have an intrinsic contribution resulting from Berry curvature. Importantly, we observed a large THE in the system arising from changes in the band structure caused by spin canting, further supported by DFT calculations. Synthesis of high‐quality single crystals of the FM phase of EuCd_2_As_2_ is quite simple, and it should motivate other researchers to study its other exotic quantum phenomena, such as the anomalous thermal Hall effects and quantum anomalous Hall (QAH) effect. This discovery opens avenues for studying more MWSMs in order to theoretically and experimentally creates more ideal WSMs with anomalous electrical and thermal transport.

## 4 Experimental Section

### Single‐Crystal Growth of EuCd_2_As_2_ and Characterizations

The single crystals of FM‐EuCd_2_As_2_ were grown by using salt‐flux (equimolar mixture of NaCl and KCl) method. As‐purchased high‐quality elemental Eu, Cd, and As pieces were mixed with NaCl and KCl inside a glove box for the synthesis process. Eu, Cd, As, NaCl, and KCl were mixed in the molar ratio Eu:Cd:As:NaCl:KCl of 1:2:2:4:4. All starting materials were loaded in an alumina crucible that was sealed in a quartz tube under vacuum (10^−5^ Torr). The tube was heated to 742 K over 24 h, soaked for 24 h, and then slowly heated to 870 K over 15 h. It was then soaked for 24 h, followed by another heating to 1120 K, then soaked for 100 h. Finally, the tube was cooled to 773 K over 300 h and then quenched in air. Shiny plate crystals were recovered by washing the product with deionized water followed by vacuum filtration.

The single crystals of AFM‐EuCd_2_As_2_ were grown by using Sn‐flux. Eu, Cd, As, and Sn were mixed in the molar ratio Eu:Cd:As:Sn of 1:2:2:10. All starting materials were loaded in an alumina crucible that was sealed in a quartz tube under vacuum (10^−5^ Torr). The tube was heated to 1173 K over 12 h, soaked for 20 h, and then slowly cooled to 773 K over 200 h. After centrifuging at 773 K to remove excess Sn, the crystals were recovered. The composition of the EuCd_2_As_2_ crystal was examined using scanning electron microscopy with an energy‐dispersive EDAX analyzer. White‐beam backscattering Laue X‐ray diffraction was used to determine the single crystallinity of the as‐grown crystal.

### Magnetization Measurements

The magnetization measurement was performed using a Quantum Design MPMS3 instrument.

### Electrical Transport Measurements

The electrical transport properties were measured using a physical property measurement system (PPMS) instrument (ETO option, Quantum Design). For transport measurements, the samples were cut into regular rectangle shapes, and a six‐probe method was applied to simultaneously measure the ordinary resistance and Hall resistivity. The final resistivity (Hall data) was symmetrized (anti‐symmetrized) to exclude the misalignment of the electrodes.

### Nernst Thermopower Measurements

A Quantum Designed physical property measurement system (PPMS)‐9 with a breakout box was used for Nernst thermopower measurements. Experiments were conducted using a Keithley 2182A nanovolt meter and Keithley 6220 current sources to provide heater current. A 120 Ω resistance heater and a copper sheet heat spreader were used to generate a temperature gradient. An alumina heat sink was attached to the bottom of the crystal. The temperature gradient was measured with copper‐constantan thermocouples attached to the crystal at two points using Ag epoxy. A set of copper wires was attached in a direction transverse to both the applied magnetic field as well as the heat flow to collect data of the Nernst effect.

Based on the following formula, the Nernst thermopower was calculated: *S*_yx_  =  *L*_x_ *V*_y_/(*L*_y_∇*T*_x_), where distances between the two temperature leads, transverse voltage and voltage wire distance are represented by *L*_x_, *V*_y_ and *L*_y_, respectively.

### Angle‐Resolved Photoemission Spectroscopy

The ARPES experiments were carried out by soft X‐ray ARPES (SX‐ARPES) at the ADRESS beam line of the Swiss Light Source^[^ ^61^ ^]^ with a PHOIBOS‐150 analyzer.^[^ ^62^ ^]^ The single crystal samples were cleaved in‐situ at 15 K. The base pressure was below 1 × 10^−10^ mbar. The data were collected using photon energies in the soft X‐ray regions, with an overall energy resolution on the order of 50–80 meV.^[^ ^30^ ^]^

### Scanning Tunneling Microscopy

EuCd_2_As_2_ single crystals were cleaved in ultrahigh vacuum (UHV) at 77 K, and immediately inserted into the scanning tunneling microscope head, held at 4.3 K. The topographic images were taken in constant current mode using electrochemically etched W‐tips. d*I*/d*V* conductance maps for quasiparticle interference were collected using a standard lock‐in technique with a 4‐mV peak‐to‐peak modulation at a frequency of 997.3 Hz.

### Computational Methodology

The electronic structure calculations were performed on the basis of DFT using the plane‐wave projected augmented wave (PAW) method as implemented in the Vienna ab initio Simulation Package (VASP).^[^ ^63^, ^64^, ^65^ ^]^ For self‐consistent calculations, a 11 × 11 × 7 k‐points mesh was used. This choice of the k‐mesh and a plane‐wave cutoff of 550 eV were found to provide a good convergence of the total energy. The Perdew–Burke–Ernzerhof (PBE)^[^ ^66^ ^]^ exchange correlation functional within the GGA was used. The electron–electron correlation effects beyond GGA at the magnetic Eu 4*f* site were taken into account by the GGA*+U* method,^[^ ^67^ ^]^ where the value of the onsite Coulomb interaction strength *U* was chosen to be 5 eV. Employing the Wannier interpolation technique,^[^ ^68^ ^]^ the intrinsic Berry curvature contribution to the anomalous Hall conductivity was assessed. To compute the Berry curvature, the MLWFs Hamiltonian projected from the GGA*+U+SOC* Bloch wave functions using the Wannier90 tool was first constructed.^[^ ^69^, ^70^, ^71^ ^]^ Atomic orbital‐like MLWFs of Eu*‐s, d, f*; Cd*‐s, p*; and As*‐p* states were considered to construct the tight‐binding Hamiltonian, which reproduced the spectrum of the system accurately in the energy window of ± 1 eV around the Fermi energy. With the tight‐binding model Hamiltonian, the intrinsic AHC using the linear response Kubo formula approach was calculated, as follows:

(1) Ωn,ijk⇀=Im∑m≠nnk⇀υ^imk⇀mk⇀υ^jnk⇀−i↔jεn−εm2

where $\langle m(\overset{⇀}{k})|$ and $\langle n(\overset{⇀}{k})|$are the eigenstates, *ε* are the eigen energies of the Hamiltonian *H*, and ${\hat{\upsilon }}_{i}$ is the velocity operator.

Subsequently, the AHC by was calculated:

(2) $$\sigma_{H}^{A}=-\frac{e^{2}}{\hbar }\sum_{n}\int \frac{dk}{{\left(2\pi \right)}^{3}}\Omega_{n,\mathrm{xy}}\left(k\right)$$

A k‐point mesh of 300 × 300 × 300 for the calculation of the AHC using Equation (2) was used.

For the surface Fermi arc calculations, the surface Green's function method as implemented in the WannierTools package was used.^[^ ^72^, ^73^, ^74^ ^]^

## Conflict of Interest

The authors declare no conflict of interest.

## Supporting information

Supporting Information

## Data Availability

The data that support the findings of this study are available from the corresponding author upon reasonable request.
