# Supplementary material for: Anomalous Hall Conductivity and Nernst Effect of the Ideal Weyl Semimetallic Ferromagnet EuCd2As2
Source: Adv Sci (Weinh). 2023 Feb 24;10(13):2207121. doi: 10.1002/advs.202207121 (PMC10161038; doi:10.1002/advs.202207121)
Supplement: Supplementary file 1 — Supporting Information [file ADVS-10-2207121-s001.pdf]

ADVANCED SCIENCE
Open Access

# Supporting Information

for *Adv. Sci.*, DOI 10.1002/advs.202207121

Anomalous Hall Conductivity and Nernst Effect of the Ideal Weyl Semimetallic Ferromagnet $EuCd_2As_2$

*Subhajit Roychowdhury*, Mengyu Yao, Kartik Samanta, Seokjin Bae, Dong Chen, Sailong Ju, Arjun Raghavan, Nitesh Kumar, Procopios Constantinou, Satya N. Guin, Nicholas Clark Plumb, Marisa Romanelli, Horst Borrmann, Maia G. Vergniory, Vladimir N. Strocov, Vidya Madhavan, Chandra Shekhar and Claudia Felser**

# ***Supporting Information (SI)***

# Anomalous Hall conductivity and Nernst effect of the ideal Weyl semimetallic ferromagnet $EuCd_2As_2$

*Subhajit Roychowdhury*[1, ‡,*], *Mengyu Yao*[1, ‡], *Kartik Samanta*[1, ‡], *Seokjin Bae,*[2] *Dong Chen,*[1] *Sailong Ju*[3], *Arjun Raghavan*[2], *Nitesh Kumar*[1,4], *Procopios Constantinou*[3], *Satya N. Guin*[1,5], *Nicholas Clark Plumb*[3], *Marisa Romanelli*[2], *Horst Borrmann*[1], *Maia G. Vergniory*[1,6], *Vladimir N. Strocov*[3], *Vidya Madhavan*[2], *Chandra Shekhar*[1], *& Claudia Felser*[1,*]

[1]*Max Planck Institute for Chemical Physics of Solids, 01187 Dresden, Germany*
[2]*Department of Physics and Materials Research Laboratory, University of Illinois Urbana-Champaign, Urbana, IL, USA*
[3]*Swiss Light Source, Paul Scherrer Institute, CH-5232, Villigen-PSI, Switzerland*
[4]*S. N. Bose National Centre for Basic Sciences, Salt Lake City, Kolkata 700 106, India*
[5]*Department of Chemistry, Birla Institute of Technology and Science, Pilani- Hyderabad Campus, Hyderabad 500078, India*
[6]*Donostia International Physics Center, 20018 Donostia-San Sebastian, Spain*
**E-mail: subhajit.roychowdhury@cpfs.mpg.de; Claudia.Felser@cpfs.mpg.de*
[‡]These authors contributed equally

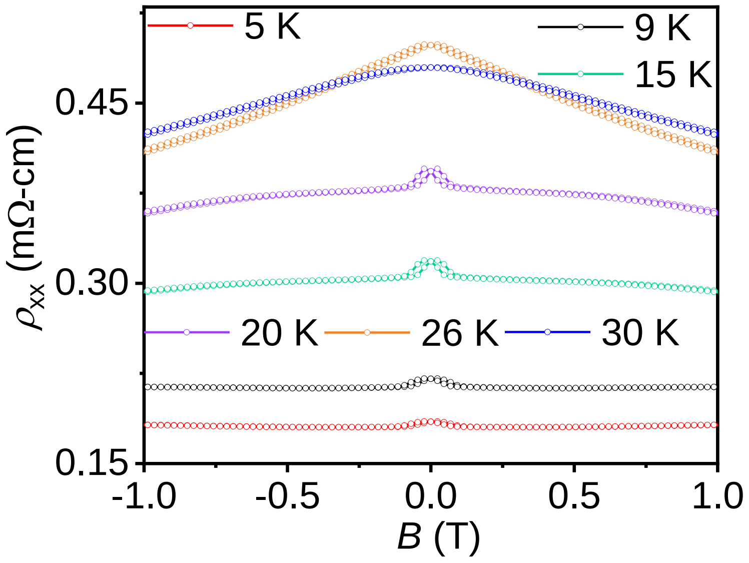


**Fig. S1:** Field-dependent resistivity ($\rho_{xx}$) of $EuCd_2As_2$ at various temperatures.

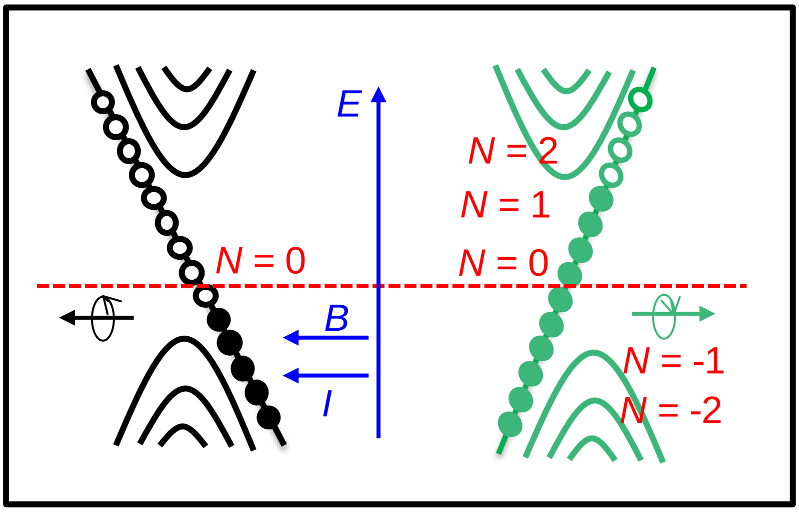


**Fig. S2:** Illustration of the chiral anomaly.

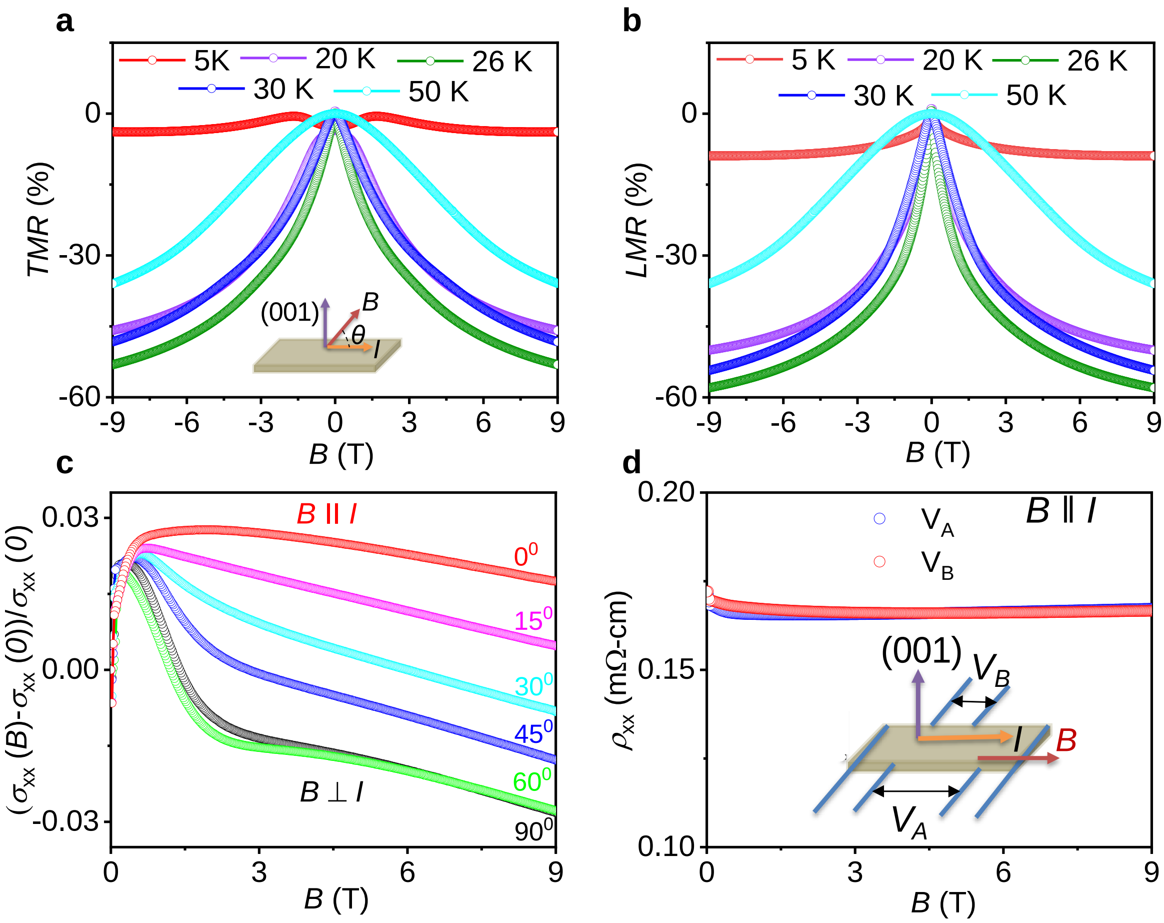


**Fig. S3:** Field-dependent **a** TMR and **b** LMR at different temperatures. Inset of fig. **a** represents the measurement configuration. **c.** Field dependence of the magnetoconductivity at different angles between the magnetic field, *B* and electrical current direction, *I*. **d.** Field dependent resistivity for two different voltage contact at *B* // *I* configuration. The insets show the schematic of the measurement set-up.

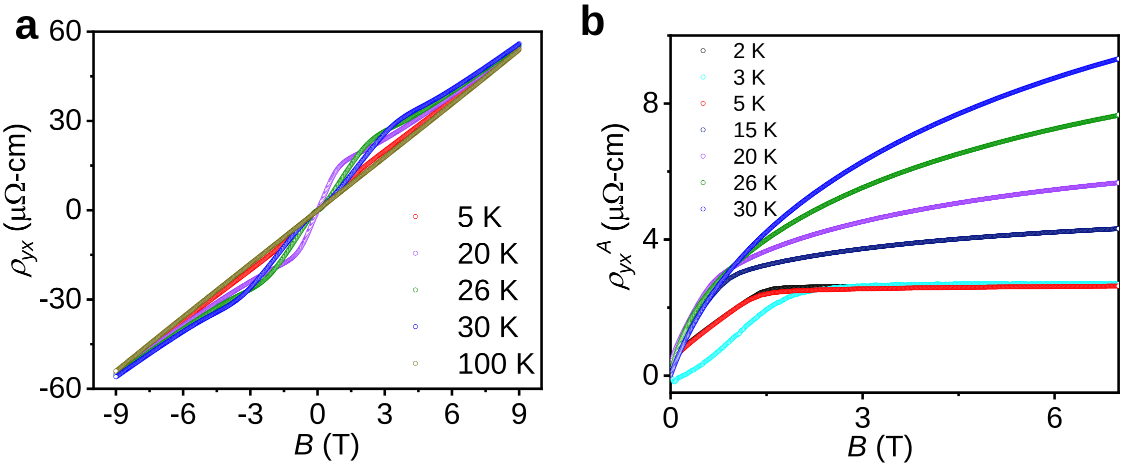


**Fig. S4:** Field-dependent **a** Hall resistivity ($\rho_{\text{yx}}$), and **b** anomalous Hall resistivity ($\rho_{yx}^{A}$) at various temperatures.

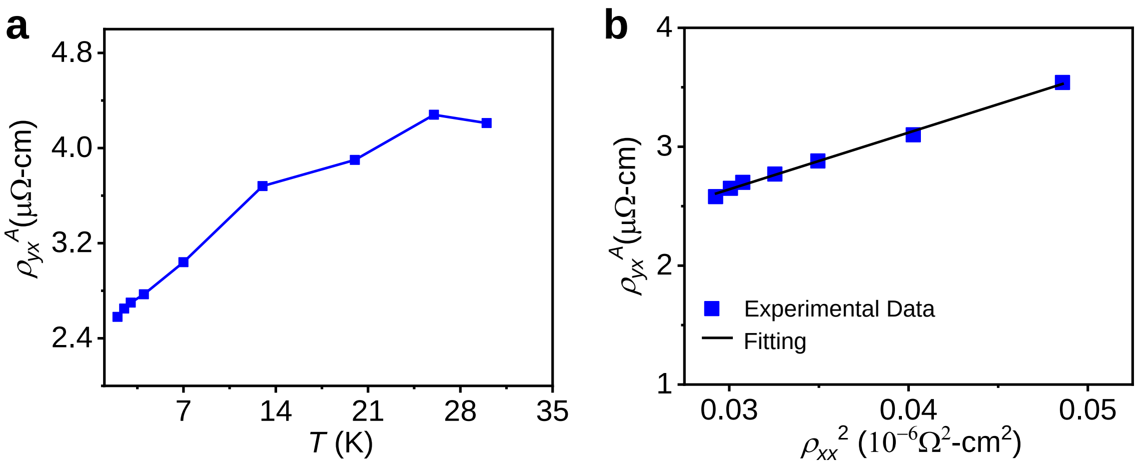


**Fig. S5: a.** Temperature dependent anomalous Hall resistivity. **b.** Scaling relation between the anomalous Hall resistivity ($\rho_{yx}^{A}$) to resistivity ($\rho_{xx}$) for $EuCd_2As_2$.

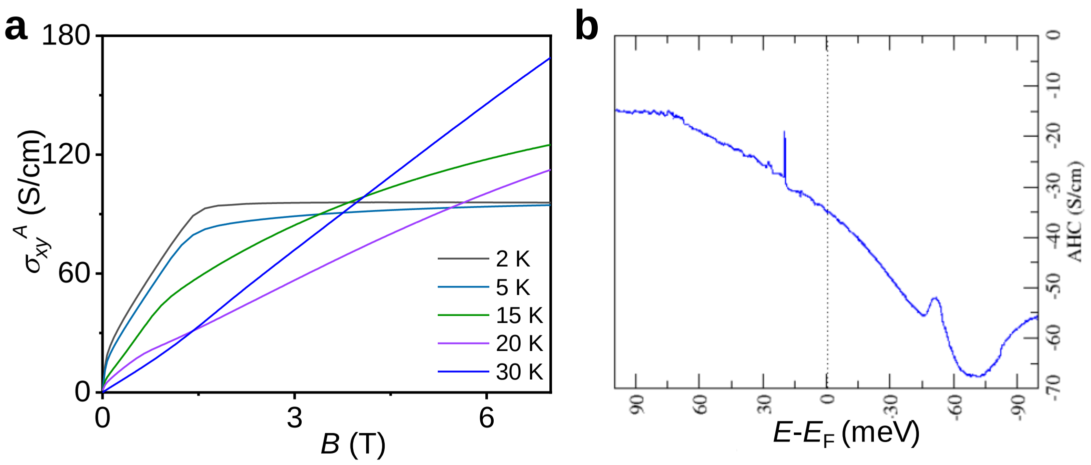


**Fig. S6: a** Field-dependent anomalous Hall resistivity ($\sigma_{xy}^{A}$) at various temperatures. b. Theoretically calculated Hall conductivity as a function of chemical potential.

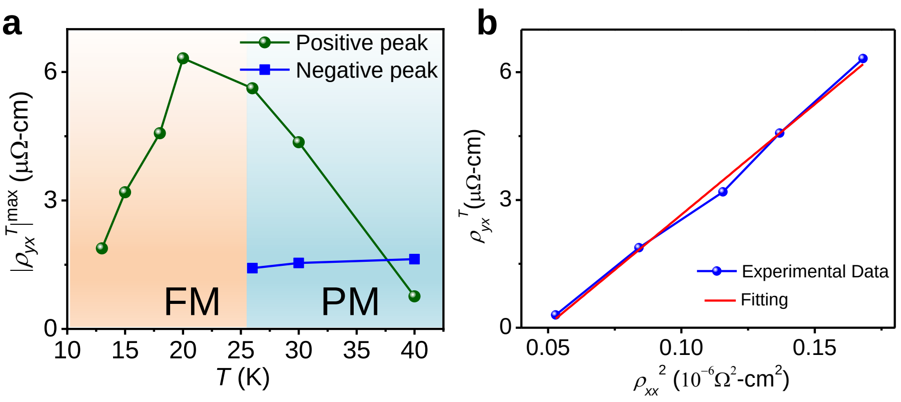


**Fig. S7**: **a.** Temperature dependent maximum $\rho_{yx}{}^{T}$ (absolute values) in the ferromagnetic (FM) and paramagnetic (PM) region. **b.** Scaling relation between the topological Hall resistivity ($\rho_{yx}^{T}$) to resistivity ($\rho_{xx}$) for $EuCd_2As_2$.

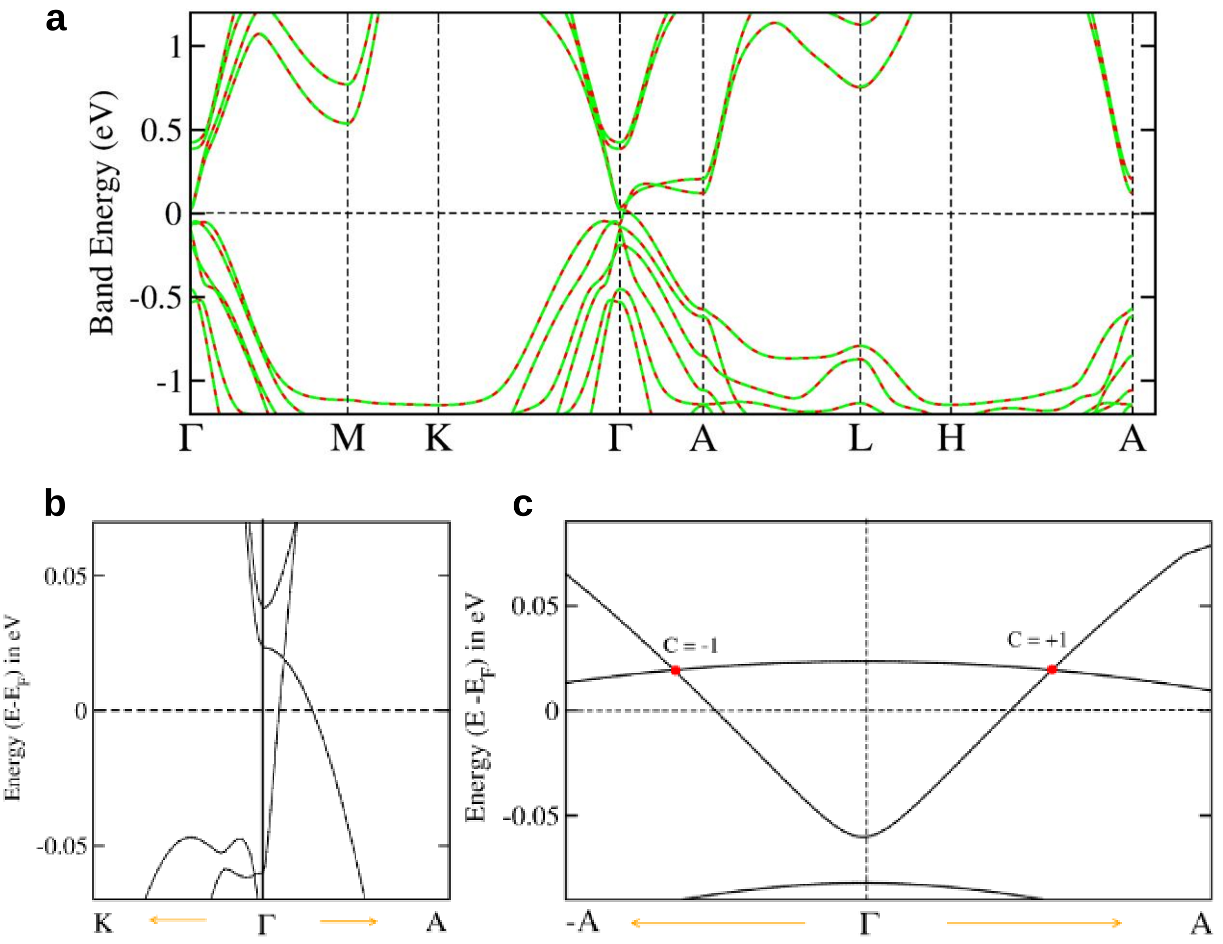


**Fig. S8:** **a** Band structure of $EuCd_2As_2$ in *GGA* + *U* + *SOC* for the FM state with magnetic moment along the *c* axis. Green lines: *GGA* + *U* + *SOC* first principles electronic bands. Red lines: Wannier-interpolated Cd-*s* + *d* + *f*, Cd-*s* + *p*, and As-*p* band structure. **b** *GGA* + *U* + *SOC* band structure band structure along K-Γ-A path. **c** Single pair of WPs around the Γ point (A-Γ-A path).

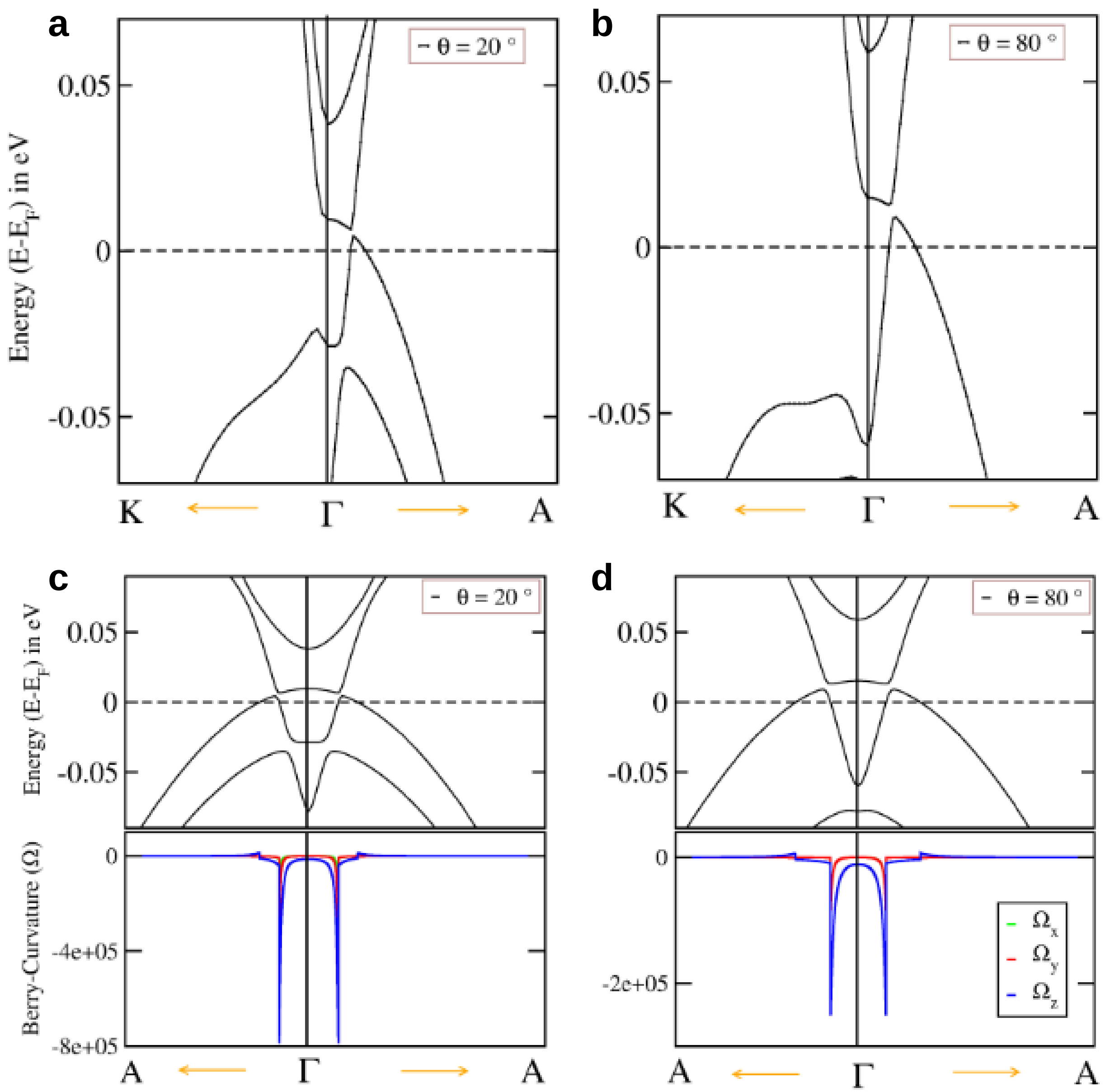


**Fig. S9:** Band structure of ferromagnetic (FM) $EuCd_2As_2$ in GGA+U+SOC, zoomed along the (K-Γ-A) high symmetry line of the hexagonal Brillouin zone for magnetic moment along **a.** the *c*-axis i.e. $\theta$=$20^0$, **b**. $\theta$=$80^0$ canting. **c-d**. Ferromagnetic band structure of $EuCd_2As_2$ zoomed into the locations of the Weyl point in comparison with the computed berry curvature along the high symmetry line of A – Γ- A for the magnetic moment along **(c)** the *c*-axis i.e. $\theta$ = $20^0$, **(d)** $\theta$=$80^0$ canting.

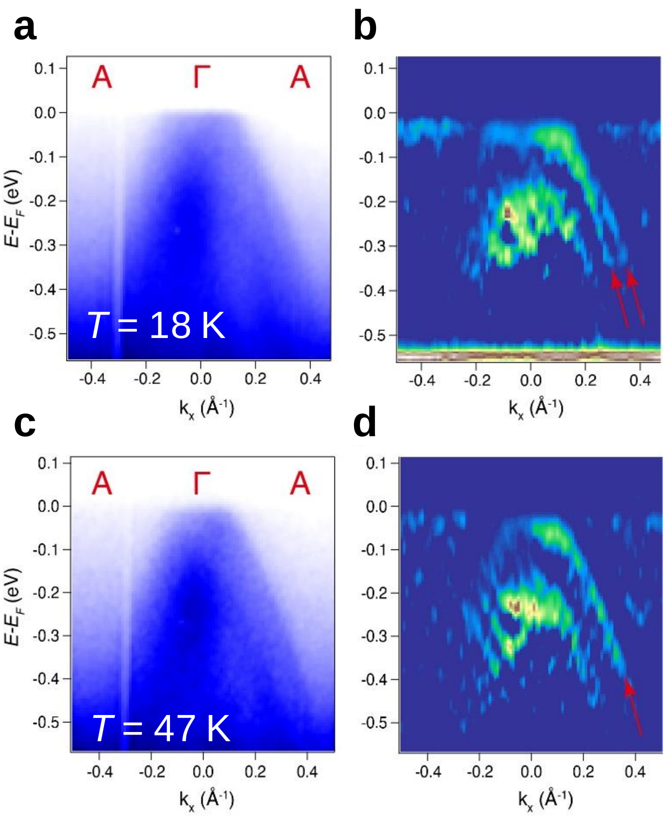


**Fig. S10:** ARPES intensity and corresponding curvature plots along the *Γ*–*A* direction measured **a,b** at 18 K and **c,d** 47 K, acquired with $h\nu$ = 250 eV

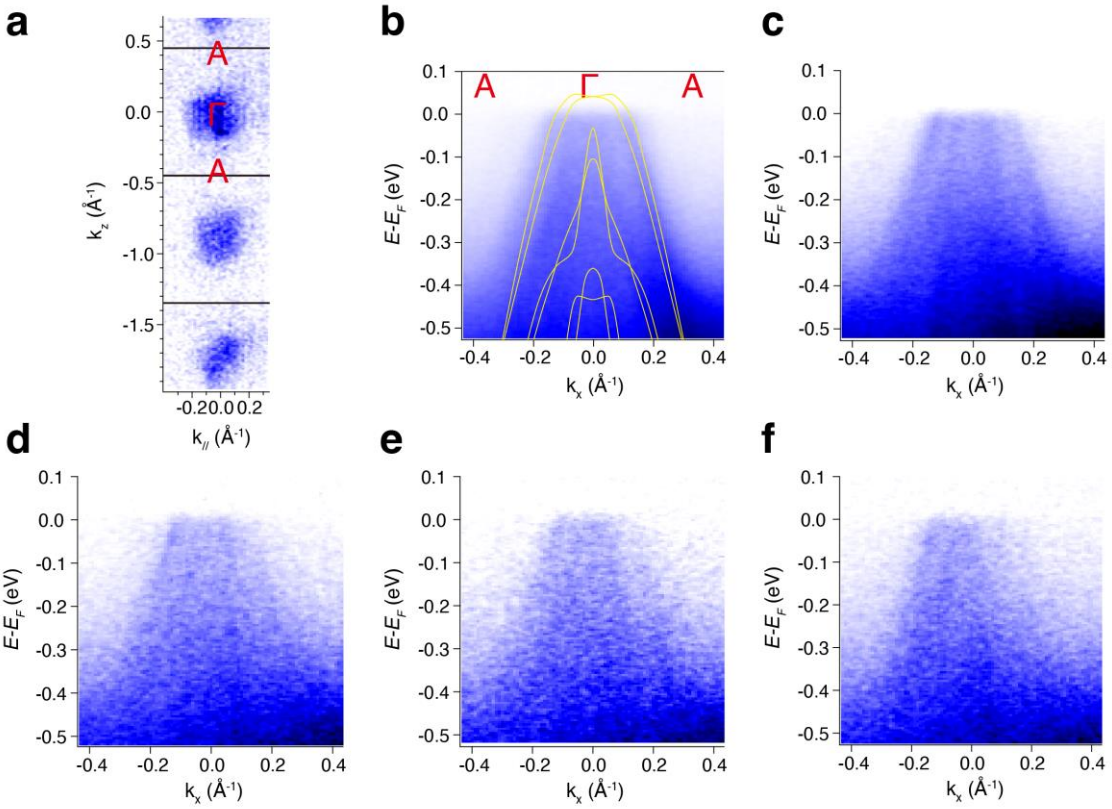


**Fig. S11: a.** Fermi surface mapping in $k_{//}$-$k_z$ plane. **b.** ARPES intensity plot overlaid with the calculated band structure. The ARPES spectrum is acquired along A-Γ-A direction at 18 K with 126 eV and C+ polarized photon. **c.** same with (b), except for acquired with LH polarized photon. **d-e.** same with (c), except for after potassium dopant of **d.** 6 Amp x 1 min, **e.** 6 Amp x 5 min, and **f.** 6.3 Amp x 3 min.

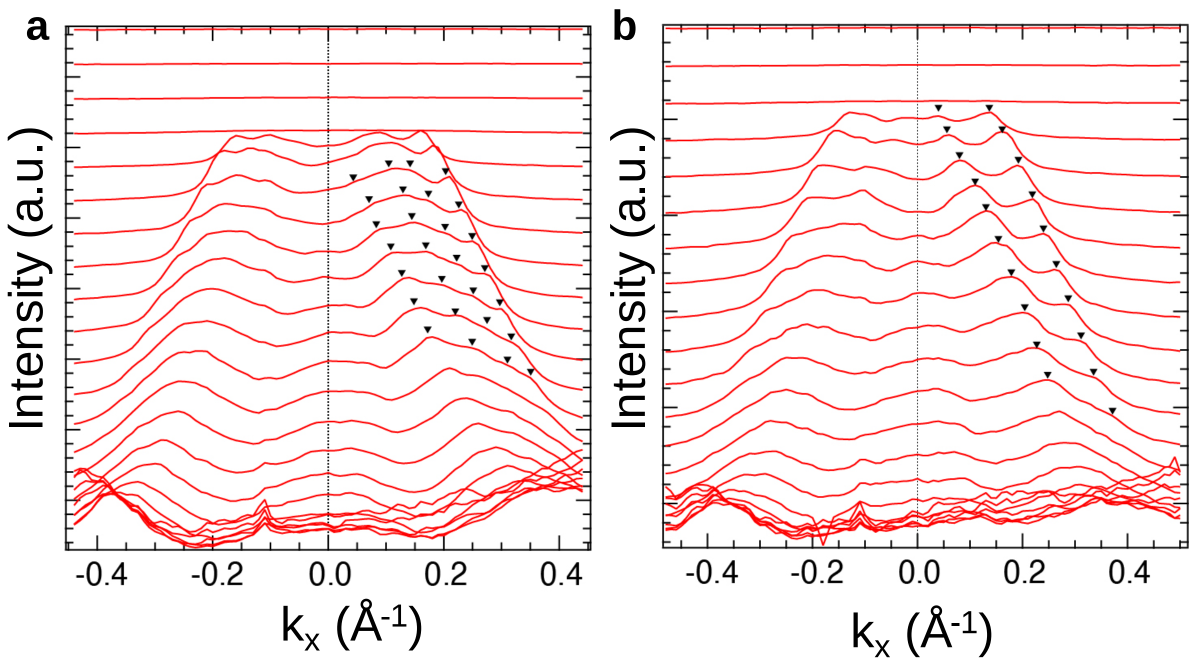


**Fig. S12: a-b.** Momentum-distribution curves of figures 3e and 3f, respectively in the main text.

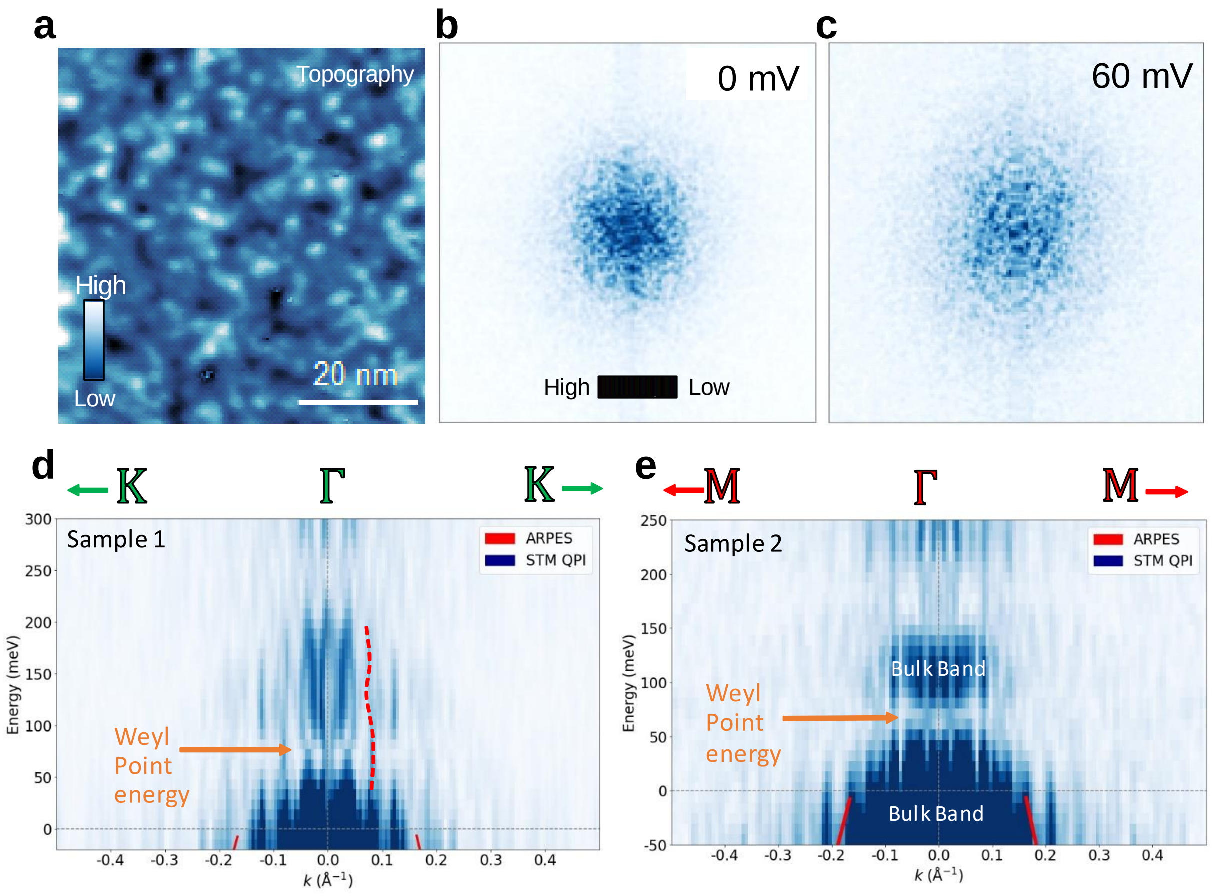


**Fig. S13: a.** 60 nm × 60 nm topographic image at 300 mV and 200 pA at the area where the dI/dV map was taken for sample 1 (the same sample used for Fig. 3**g-i** in the main text). **b-c.** Representative Fourier transforms of dI/dV conductance maps near the Γ point at 0 mV and 60 mV, respectively. **d-e.** Energy-momentum linecut of the QPI taken from (**d**) sample 1 along the Γ-*K* and (**e**) sample 2 along the Γ-M directions. The filled region of the QPI signal originates from the scattering from the bulk bands, showing their dispersion. The red solid curve is the dispersion along the Γ-M obtained from ARPES data (Fig. 3e of the main text). The orange arrow displays the expected Weyl point energy. The red dash line in (**d**) points out possible existence of the surface states which connects conduction and valence bands.

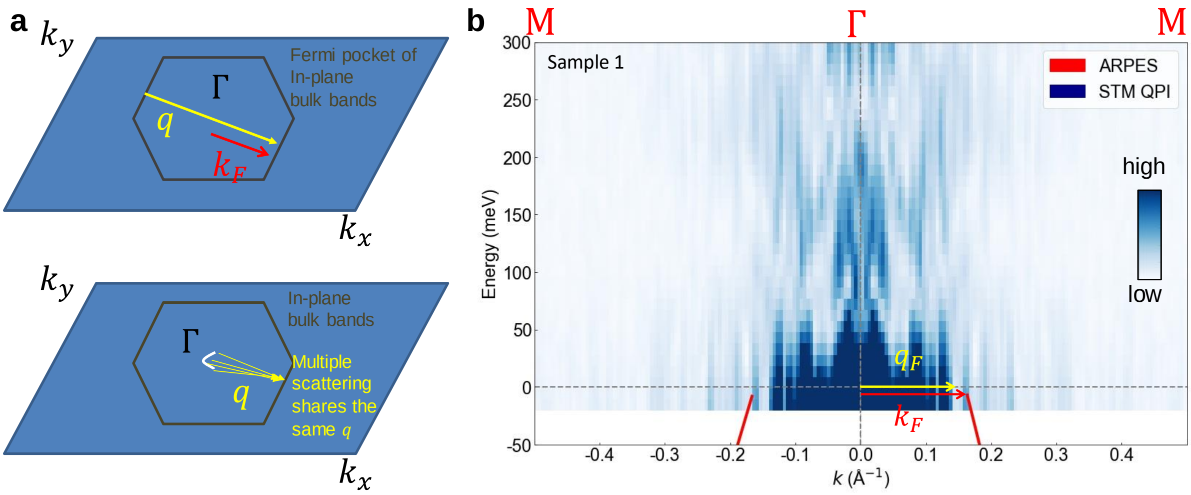


**Fig. S14: a.** Upper panel: In-plane bulk-to-bulk bands scattering; lower panel: Bulk band-to-arc states scattering. The white curve represents the projected arc states to $k_{xy}$ plane. **b.** Energy-momentum linecut of the quasiparticle interference (QPI) along the Γ-M. The dark blue intensity plot represents QPI signal and the red solid curve is the dispersion from the ARPES data along the same direction (Fig. 3e, main text). The yellow arrow represents dominant the scattering wavevector at Fermi energy ($q_F$) which is similar in the length to the Fermi wavevector ($k_F$) displayed in the red arrow.

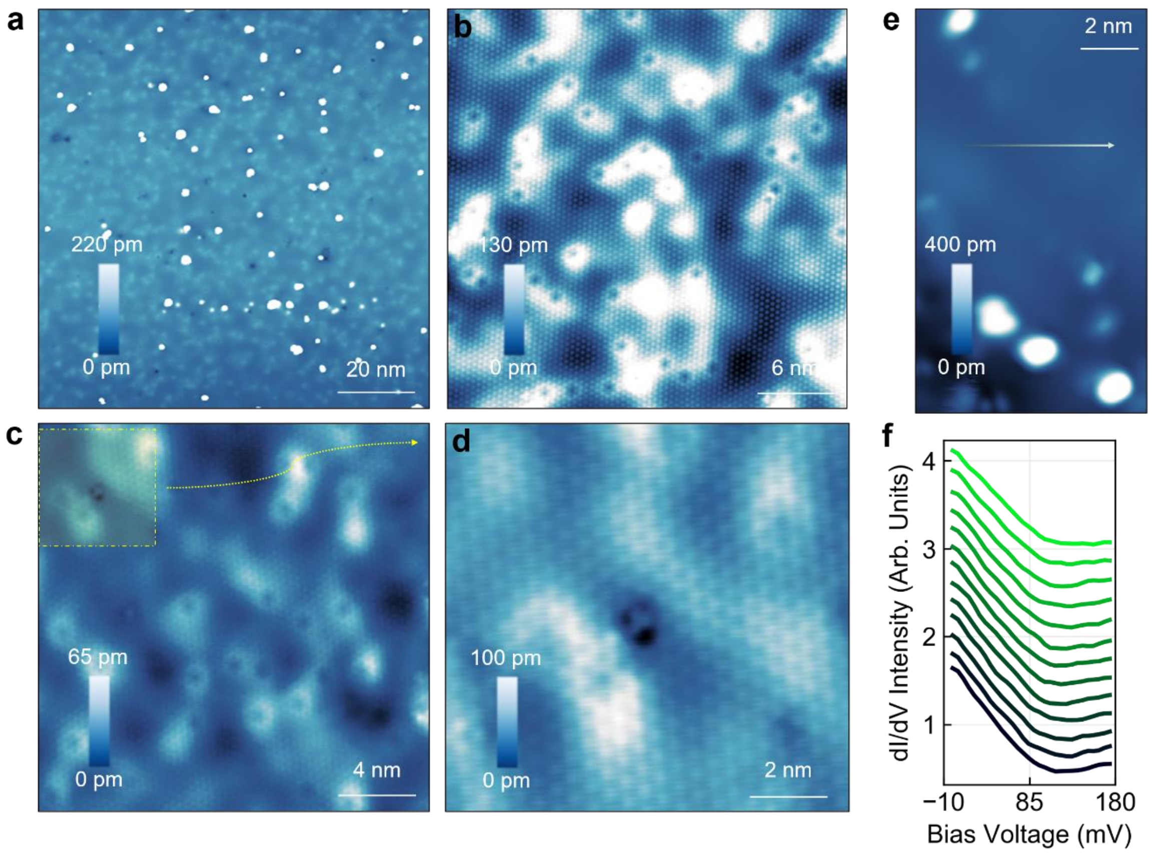


**Fig. S15:** **a**. 100 nm × 100 nm large-area topographic image of $EuCd_2As_2$ at -800 mV and 10.0 pA. **b**. 30 nm × 30 nm topographic image at -20 mV and 1.00 nA showing a hexagonal lattice with 4.44 Å spacing. **c**. 30 nm × 30 nm topographic image at 60 mV and 1.00 nA showing surface with several types of defects. **d**. 10 nm × 10 nm part of the larger area topographic image in (**c**) with a close-up of one type of surface defect. **e**. 8 nm × 14 nm topographic image of a bulk region at -400 mV and 60.0 pA on which dI/dV spectra are taken. **f**. dI/dV spectra linecut along the arrow shown in (**e**) showing spatial consistency in spectral features with a minimum density of states in the 100-120 meV energy range.

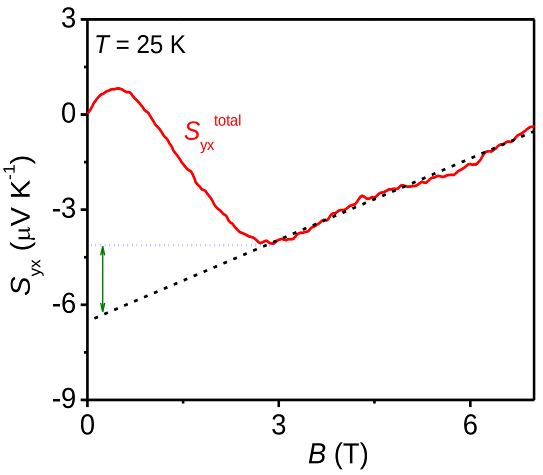


**Fig. S16:** Field dependent Nernst thermopower ($S_{yx}$) at 25 K to extract the uncertainty in the anomalous part of the Nernst thermopower.

The Nernst thermopower has been estimated from the following ways. Since the Nernst thermopower shows contributions from both ordinary and anomalous Nernst thermopower like the Hall data. Therefore, The total measured Nernst thermopower, $S_{yx}$ in a magnetic system is usually defined as a combination of two terms: $S_{yx} = S^{O} + S^{A}$, in which the ordinary part $S^{O}$ is proportional to the applied magnetic field. To determine the anomalous Nernst thermopower $S^{A}$, a linear fitting from high field to zero-field is extrapolated, then a non-zero value of Nernst thermopower at zero field is determined. Indeed, the cut at zero field is completely dependent on the slope of ordinary Nernst thermopower. In such determination procedure, the value of $S^{A}$ is either over or under estimated depending on slope of high field data.

Our value is over estimated from the aforementioned procedure since $S^{O}$ contains a finite slope. To minimise the error, we have considered a maximal method as given in the Fig. S16. The minimum value of $S_{yx}^{A}$ is 4.2 μV/K as measured from the dip assuming an artificial saturation for further field. However, $S_{yx}^{A}$ is 6.4 μV/K, which is the maximum as measured from the high field slope (a common method which is generally applied). Between these two values, there is error of 20%, which is acceptable in such cases. We have compared the anomalous Nernst thermopower for FM-$EuCd_2As_2$ with other reported ferromagnets and non-collinear antiferromagnets in fig.5 with estimated $S_{yx}^{A}$ ~5.3 ± 1.1 μV/K.

**TABLE S1.** Parameters determined from refinements of room temperature X-ray diffraction data taken on single crystals of AFM and FM $EuCd_2As_2$. Space group *P-3m1* (no.164), Eu in 1*a* (0,0,0); Cd and As in 2 *c* (0,0,*z*). Occupancy was refined individually for each site but kept at unity in final refinement in case deviation turned out insignificant. $U_{eq}$ values are given as derived from anisotropic model.

| **Parameters** | **AFM- $EuCd_2As_2$** | **FM- $EuCd_2As_2$** |
|---|---|---|
| *a* (Å) | 4.4482(3) | 4.4437(4) |
| *c* (Å) | 7.3413(8) | 7.3313(11) |
| $\alpha$ (°) | 90 | 90 |
| $\beta$ (°) | 90 | 90 |
| $\gamma$ (°) | 120 | 120 |
| V (Å$^3$) | 125.80(2) | 125.37(3) |
| Eu site occupancy | 0.996(3) | 0.990(2) |
| Cd site occupancy | 1.005(4) | 1.000(3) |
| As site occupancy | 1.000(4) | 1.000(3) |
| $z_{Cd}$ | 0.36667(5) | 0.36696(5) |
| $z_{As}$ | 0.75315(6) | 0.75319(7) |
| $U_{Eu}$ (Å$^2$) | 0.01084(9) | 0.01121(12) |
| $U_{Cd}$(Å$^2$) | 0.01299(9) | 0.01329(11) |
| $U_{As}$(Å$^2$) | 0.00947(10) | 0.01004(12) |

CCDC contains a full set of crystallographic data for these structures. The data can be obtained free of charge from The Cambridge Crystallographic Data Centre via www.ccdc.cam.ac.uk/structures.

**Table S2:** Position of Weyl point with canting angle.

| **Band** | **Coordinates** | | | **Position of WP from $E_F$ (meV)** | **Chirality** | **Canting angle ($^0$)** |
|---|---|---|---|---|---|---|
| | **$k_1$** | **$k_2$** | **$k_3$** | | | |
| 19 | 0 | 0 | 0.025 | 1.87 | -1 | 10 |
| 19 | 0 | 0 | -0.025 | 1.87 | +1 | |
| 19 | 0 | 0 | 0.026 | 5.68 | -1 | 20 |
| 19 | 0 | 0 | -0.026 | 5.68 | +1 | |
| 19 | 0 | 0 | 0.026 | 11.22 | -1 | 80 |
| 19 | 0 | 0 | -0.026 | 11.22 | +1 | |
| 19 | 0 | 0 | 0.027 | 19.31 | -1 | 90 |
| 19 | 0 | 0 | -0.027 | 19.31 | +1 | |
